# Immunoregulation by ESAT-6: From Pathogenesis of Tuberculosis to Potential Anti-Inflammatory and Anti-Rejection Application

**DOI:** 10.3390/ph18091408

**Published:** 2025-09-18

**Authors:** Weihui Lu, Jingru Lin, Yuming He, Bin Yang, Feifei Qiu, Zhenhua Dai

**Affiliations:** 1 Section of Immunology, the Second Affiliated Hospital of Guangzhou University of Chinese Medicine, Guangzhou 510006, China; weihui.lu@gzucm.edu.cn (W.L.);; 2 Department of Cardiovascular Sciences, College of Life Sciences, University of Leicester, Leicester LE1 9HN, UK; by5@le.ac.uk

**Keywords:** anti-inflammatory drug, ESAT-6, immunoregulation, transplant rejection, immune cells

## Abstract

The early secreted antigenic target of 6 kDa (ESAT-6), a main effector molecule of the ESX-1 secretion system, is identified as a virulence determinant and immunoregulatory protein of *Mycobacterium tuberculosis* (Mtb), affecting the interaction between host immune cells and pathogens. ESAT-6 facilitates the survival of mycobacteria and their cell-to-cell spreading through membrane-permeabilizing activity and the regulation of host immune cell functions. In this review, we first summarize the recent knowledge of the roles of ESAT-6 in the survival of bacteria, phagosomal escape, and pathogenicity during Mtb infection. Then, we focused on its complex immunomodulatory effects on different immune cells, such as macrophages, dendritic cells, neutrophils, and T cells, accentuating its capability to either facilitate or inhibit immune responses through different signaling pathways. While our review has summarized its main roles in immunopathology in the context of tuberculosis, we additionally search for emerging evidence indicating that ESAT-6 has anti-inflammatory and immunosuppressive properties. Particularly, we discuss recent preclinical studies showing its capability to suppress transplant rejection and alloimmunity, probably via the induction of regulatory T cells. Nevertheless, the potential clinical use of ESAT-6 remains uncertain and needs further verification by comprehensive preclinical and clinical studies. Thus, we propose that ESAT-6 may be exploited to ameliorate immunopathology in TB infection and to suppress immune-mediated inflammation or transplant rejection as well.

## 1. Introduction

Early secreted antigenic target of 6 kDa (ESAT-6) with a gene name of EsxA is a small secreted protein of *Mycobacterium tuberculosis* (Mtb), which is an infectious agent worldwide and causes tuberculosis [1]. ESAT-6 is observed in the isolates of Mtb and *Mycobacterium bovis* (*M. bovis*), while it is not detected in all of the attenuated sub-strains of *M. bovis* BCG, such as Danish 1331, Tokyo, Moreau, Russia, Glaxo, Pasteur, Canadian, and Tice, and less virulent *M. microti* resulting from the lack of a gene encoding ESAT-6 [1,2]. The gene encoding ESAT-6 protein, RV3875, is located in a specific genetic locus known as region of difference (RD)1, which is absent in all BCG strains and harmless *M. microti* but present in the pathogenic *M. bovis* and Mtb [1,3,4].

ESAT-6 is a polypeptide of 95 amino acids that are highly conserved among mycobacterium, including *M. tuberculosis* (H37RV), *M. bovis*, *M. kansasii*, *M. marinum*, *M. smegmatis*, and *M. leprae* [5]. Structural studies and gene analyses have demonstrated the loss of a traditional secretion signal in ESAT-6 protein, leading to the identification of a specific secretion system named as ESX-1, which is responsible for the release of this protein [6]. ESX-1 consists of several genes (Rv3866-Rv3883c) [7] located in the RD1 region [8], while proteins encoded by these genes possibly form a multi-subunit cell-envelope-spanning structure for the export of ESAT-6 [6]. Studies using gene deletion and reintroduction of RD1 have shown its important role in the virulence of Mtb [9,10]. Pym et al. reported that the vaccine BCG complemented with the RD1 locus secreted ESAT-6 and caused specific immune responses depending on this antigen [11]. Further investigations by other groups revealed that individual genes located within RD1 also had an effect on the secretion of ESAT-6 protein [12,13].

Many studies have made significant progress in understanding the mechanisms responsible for the roles of ESAT-6 in Mtb pathogenesis or immunopathology, and these works have been previously reviewed by other groups [5,14,15,16,17]. However, recent studies have demonstrated that ESAT-6 exerts anti-inflammatory effects and that it can suppress alloimmunity or transplant rejection [18]. Here, we generalize the recent advances in investigations into ESAT-6 and its roles in immunoregulation while focusing on some of the key questions for futuristic study and, in particular, its potential application based on its anti-inflammatory and immunosuppressive properties.

## 2. ESAT-6 Is a Virulence Determinant for Mtb

An early study proved that ESAT-6, as well as CFP-10, was vital for Mtb virulence and survival. The authors showed that these two proteins contributed to the fusion of lysosomes and phagosomes containing Mtb, followed by phagosome rupture and translocation of Mtb from phagolysosomes to the cytosol in host cells, favoring mycobacteria replication and spread [19].

Recent works have pinpointed that ESAT-6 has membrane-permeabilizing activity, which is related to cytosolic translocation and virulence of Mtb. Investigators demonstrated that mutations at glutamine 5 or post-secretion modification of ESAT-6 protein changed its membrane-permeabilizing activity, subsequently affecting the virulence and cytosolic translocation of Mtb and Mycobacterium marinum in murine macrophages and zebra fish embryos [20,21]. Nα-acetylation of the Thr-2 residue on ESAT-6, which is exclusively presented in mycobacteria and promotes disassociation of ESAT-6 and EsxB heterodimer at low pH, is a precondition for the interaction of ESAT-6 with the host cell membrane, resulting in increased mycobacterial cytosolic translocation and virulence [22].

Lipid body accumulation in cells caused by an Mtb infection resulted in the development of foamy macrophage (FM), which provided abundant nutrients for mycobacteria and protected them from bactericidal activities [23,24,25]. Enhanced glucose uptake with metabolic flux perturbations then induced the differentiation of macrophages into FMs [24]. ESAT-6 was identified as a regulator of FM formation through the mediation of the processes mentioned above. ESAT-6 strengthened glucose uptake mediated by GLUT-1 and disturbed the glycolytic pathway in macrophages, accompanied by the accumulation of DHAP required for Triglyceride synthesis as well as AcCoA for the synthesis of 3-HB [26].

## 3. ESAT-6 Plays an Immunoregulatory Role

As a result of evolved mechanisms, Mtb has an abundant repertoire of antigen molecules to change the host immune system, including innate and adaptive immunity, and to promote infection. ESAT-6, one of such antigen molecules, exhibits strong immunoregulatory effects on several immune cells through different molecular and signal pathways (Table 1 and Table 2).

### 3.1. Effects of ESAT-6 on Innate Immunity

#### 3.1.1. Macrophages

Macrophages play a role in the immune system and carry out different functions such as the phagocytosis and digestion of microorganisms, the clearance of debris and dead cells, and immunoregulation [62]. Macrophages in the lung are the primary immune cells Mtb meets when it enters the host [16]. The interaction between Mtb and macrophages is vital for a successful Mtb infection. Although Mtb is engulfed by macrophages, it can suppress their intracellular killing and antigen presentation via secreting ESAT-6, leading to the elusion of the host immune system and the establishment of a persistent infection [63].

**Figure 1:**
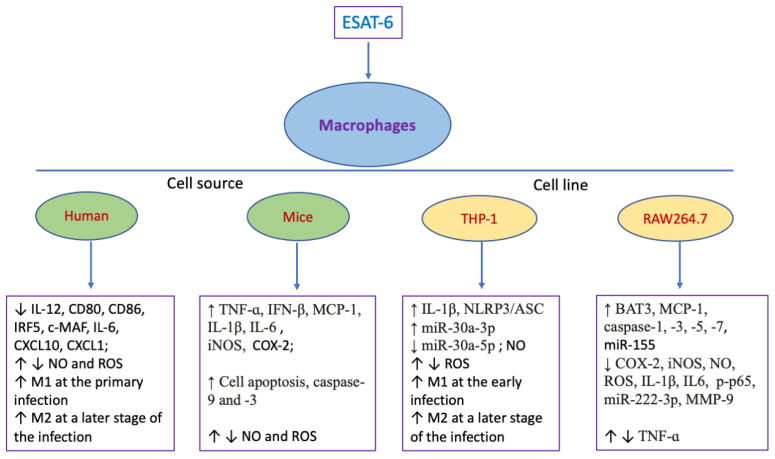
The dual regulatory effects of ESAT-6 on macrophages. As shown in this figure above, ESAT-6 plays a dual role in regulating the activation and polarization of macrophages. On one hand, ESAT-6 increases the production or expression of many proinflammatory cytokines by macrophages and promotes their M1 phenotypes at the early stage of the infection. On the other hand, it can reduce NO, ROS, and COX-2 while enhancing macrophage apoptosis and M2 polarization. (“↑” denotes increasing, while “↓” indicates decreasing).

ESAT-6 can directly interact with macrophages, allowing it to modulate the immune response of macrophages (Table 1 and Figure 1). Cyclooxygenase-2 (COX-2), known as a main mediator of inflammation, was rapidly expressed upon inflammatory and other physiological stimuli [64]. ESAT-6 promoted the production of COX-2 through PI3K and MAPK signaling axis in macrophages isolated from peritoneal exudates [54]. In addition, ESAT-6 induced the expression of TNF-ɑ and MCP-1 via the NADPH-ROS-JNK/p38-NF-κB pathway in RAW264.7 cells and through p38 MAPK signaling in bone marrow derived macrophages (BMDMs) [28,40]. Another study demonstrated that Mtb-induced IL-1β production in BMDMs was dependent on ESAT-6 [30], and that the effects of ESAT-6 on IL-1β was possibly regulated by an infection-inducible inflammasome complex containing NLRP3, ASC, and caspase-1 [33]. ESAT-6 could also significantly promote IL-6 secretion by BMDMs compared to CFP10 and antigen 85A, and this effect was dependent on STAT3 activation rather than the TLR2 pathway [29]. Furthermore, ESAT-6 was observed to enhance IFN-β expression not only in BMDMs, but also in peritoneal macrophages and MH-S cells (an alveolar macrophage cell line). This was achieved by the activation of the TLR4-TRAF signaling pathway [51]. Inducible nitric oxide synthase (iNOS), which is responsible for nitric oxide (NO) generation, plays a key role in immunologic activation and inflammation [65]. Lin et al. revealed that ESAT-6 could obviously induce iNOS/NO production and expression of epithelioid macrophage marker molecules in BMDMs, promoting the transition of macrophages into epithelioid macrophages [31]. Li et al. indicated that there is ESAT-6 augmented phagocytosis activity of THP-1 macrophages with an increase in reactive oxygen species (ROS) generation partially through the HIF1a signaling pathway [34].

On the contrary, some studies indicated that ESAT-6 played an inhibitory role in immune responses of macrophages (Figure 1). A previous study demonstrated that ESAT-6 could directly bind to TLR2, resulting in the activation of Akt and inactivation of NF-ĸB in RAW cells [46]. This study further reported that six amino acid residues at the carboxy-terminal of ESAT-6 protein were critical for TLR2-regulated repressive effects. ESAT-6 not only impeded ROS production and activity of p65 [45], but also reduced C-myc expression via regulating ERK1/2 activation [47] in the nucleus in RAW264.7 cells stimulated by LPS. Some investigators showed that ESAT-6 treatment led to decreased production of NO and ROS in not only THP-1-differentiated cells but also in murine peritoneal macrophages infected by *M. bovis* [37,49]. The double-connected structure of ESAT-6 (2E6D) attenuated expression and enzyme activity of the matrix metalloproteinase-9 (MMP-9) and suppressed COX-2, iNOS and NO in LPS-stimulated RAW 264.7 macrophages via NF-κB and MAPK pathways [44]. ESAT-6 treatment also decreased the expression of proinflammatory cytokines in macrophages and mice infected with mycobacteria Mycobacterium smegmatis through modulating miR-222-3p and its target PTEN [48]. Another study revealed that ESAT-6 induced the proinflammatory M1 phenotype with secretion of IL-6, IL-12, and TNF-ɑ and the induction of an M1 transcriptional signature in human monocyte-derived macrophages at the primary infection of Mtb, and then promoted the switch of M1 macrophages to anti-inflammatory M2 phenotypes at a late stage of the infection, helping Mtb to keep a persistent infection [27]. Recently, the effects of ESAT-6 on M1/M2 polarization were also reported by Sun et al., showing that ESAT-6 contributed to M1 polarization of THP-1 cells with the activation of the TLR4/MyD88/NF-κB pathway and cell apoptosis within 24 h, and then promoted the switch of the macrophages to M2 phenotype 36 h post-treatment followed by inactivation of the TLR4/MyD88/NF-κB pathway [38].

Autophagy, a process of intracellular degradation and recycling of their own components [66], is another mechanism by which macrophages kill invading pathogens. It was reported that ESAT-6 suppressed calcimycin-induced autophagy in THP-1 cells, which were treated with PMA, through regulating microRNA-30a, thus facilitating the intracellular survival of mycobacteria [39,67]. In both murine macrophage cell line (Raw264.7) and the primary murine macrophages, ESAT-6 prevented LC3II and SQSTM1 degradation and autophagosome-lysosome fusion through activation of mTOR signaling, leading to an increased load of bacillus Calmette–Guerin (BCG) [52]. ESAT-6 also inhibited the autophagy of macrophage cell line J774 A.1 by increasing expression and activity of SOD-2 [53].

Apoptosis, also called programmed cell death, leads to the clearance of damaged cells without eliciting inflammation [41,68]. Necrosis is an irreversible cell injury and eventual cell death that is triggered by external factors or diseases, leading to the intumescence of cell organelles, plasma membrane break, and eventual cell lysis [69]. Regarding cell apoptosis, ESAT-6 was shown to induce the apoptosis of THP-1 cells with an increase in mRNA expression of caspase genes (caspase-1, -3, -5, -7, and -8) relative to untreated cells [41]. BAT3, secreted by macrophages, could decrease production of nitric oxide and proinflammatory cytokines by macrophages stimulated with IFN-γ and LPS. Investigators demonstrated that ESAT-6 induced BAT3 release and cell apoptosis of RAW264.7 macrophages by cleavage of BAT3 [42]. In addition, ESAT-6 enhanced miR-155 expression in RAW264.7 cells through TLR2/NF-κB activation, resulting in the apoptosis of macrophages [43]. Similar effects of ESAT-6 were observed in bone marrow-derived macrophages (BMDMs). ESAT-6 induced the apoptosis of BMDMs via ROS-MAPK signaling and the activation of cleaved caspase-9 and -3 [32]. Recently, Sun et al. revealed that ESAT-6 induced cell apoptosis only presented in the proinflammatory M1-phenotype of THP-1 cells via modifying TLR4/MyD88/NF-κB axis, while it exhibited little effects on the apoptosis of anti-inflammatory M2-polarized macrophages [38]. ESAT-6 augmented NLRP3 activation and necrosis of THP-1 cells by mediating phagosomal rupture and Syk activity during Mtb infection [35,70].

ESAT-6 interacts with beta-2-microglobulin (β2M) in the host through six amino acid residues at the c-terminal region of ESAT-6 and secludes β2M in the endoplasmic reticulum, leading to the lower expression of MHC-I-β2M complexes on cell surface and inhibition of class I Ag presentation [36]. Later on, these investigators demonstrated that the interaction between ESAT-6 and β2M resulted in the sequestration of HFE protein in the endoplasmic reticulum and a decrease in expression of the HFE-TFR1 complex on the surface of macrophages, therefore promoting holotransferrin-regulated iron uptake that was vital for Mtb survival and virulence in macrophages [50].

In summary, ESAT-6 exerts a critical role in the survival and virulence of Mtb by regulating macrophages in different ways, including various inflammatory responses, autophagy, apoptosis, necrosis, and antigen presentation. Understanding of these interactions between ESAT-6 and macrophages may drive the development of new therapeutic strategies for treating tuberculosis.

#### 3.1.2. Dendritic Cells (DCs)

DCs are typical antigen presenting cells responsible for recognizing, processing, and presenting antigens to T cells [71]. ESAT-6 has been shown to play a role in regulating DCs. It was reported that ESAT-6 bound to surface receptors of DCs, such as TLR2 [55] and TLR4 [56], which induced a series of signal pathways regulating the maturation and activation of DCs [72]. Thus, ESAT-6 increased the expression of co-stimulatory molecules, such as CD80 and CD86, in bone marrow-derived DCs. Apart from its effects on DC maturation and activation, ESAT-6 also stimulated the production of proinflammatory cytokines, including IL-6, TNF-ɑ, and IL-12p40, in dendritic cells [56]. Interestingly, ESAT-6 enhanced IL-6 and TGF-β secretion by DCs through activation of TLR-2/MyD88 signaling in Mtb-infected mice, thereby strengthening Th17 cell response and differentiation [55]. However, an earlier study indicated a different regulation of ESAT-6 on DCs. In this study, human PBMCs were utilized to induce immature DCs, and then mature DCs by LPS and CD40L. The addition of ESAT-6 resulted in the inhibition of DC maturation and activation, lower levels of IL-12, higher levels of IL-1β and IL-23; thus strengthening Th17 cell response but impeding Th1 response [57].

Taken together, ESAT-6 exhibits multiple significant effects on dendritic cells, including its regulation of their function, maturation and activation, modulation of cytokine production, and induction of their apoptosis. These effects may contribute to the capacity of Mtb to evade the host immune system and establish a chronic infection.

#### 3.1.3. Neutrophils

ESAT-6 also has an impact on neutrophils. It was revealed that ESAT-6 induced the intracellular Ca(2+) overload in neutrophils, and then promoted the formation of neutrophilic extracellular traps and necrosis of phosphatidylserine-externalized neutrophils, thus helping mycobacteria escape from the antimicrobial action of neutrophils and facilitating inflammatory granuloma development vital for Mtb transmission [58].

## 4. Effects of ESAT-6 on Adaptive Immunity: T Cells

T cells are mainly divided into two large sub-lineages: αβ and γδ T cells, which are identified by the expression of αβ or γδ T-cell receptor (TCR) [73]. Normally, γδ T cells only present a small percentage of total T cells (1–5%) [74]. Most of the αβ subset of T cells are CD4+ or CD8+ cells [75]. Wang et al. observed that recombinant ESAT-6 obviously impeded the expression of IFN-γ, IL-17, and TNF-ɑ in human T cells challenged by Mtb [59]. They showed that ESAT-6 directly bound to T cells and suppressed their activation without cellular cytotoxicity or death. Similarly, another study showed that ESAT-6 inhibited IFN-γ production by human T cells upon stimulation with anti-CD3 and anti-CD28 antibodies through activation of the p38 MAPK pathway [76]. Nevertheless, an investigation by Ayman et al. indicated that short-term stimulation with ESAT-6 enhanced TNF production by CD4+ T cells and IFN-γ expression in CD8+ T cells [60]. However, ESAT-6 directly promoted activation and proliferation of human memory γδ-T cells [61]. Interestingly, another Mtb antigen, Rv2201-519, induced a robust Th1 immune response [77].

In addition to playing indirect roles through antigen-presenting cells, ESAT-6 may directly regulate T cell activation or function. It is well recognized that T cells, especially human T cells, express Toll-like receptors (TLRs), including TLR2 and TLR4 [78,79]. Given that ESAT-6 can directly bind to TLR2 and TLR4 on macrophages and DCs [55,56], it is likely that an analogous interaction may occur on T cells. Indeed, TLR2 signaling on T cells can promote effector T cell function and cytokine production [80,81]. This signaling pathway could also lead to the contrasting influences of ESAT-6 on different T cell subsets (e.g., stimulation of γδT cells [61] vs. inhibition of αβT cell responses [59,76]) and should be a focus of mechanistic research in the future.

**Figure 2:**
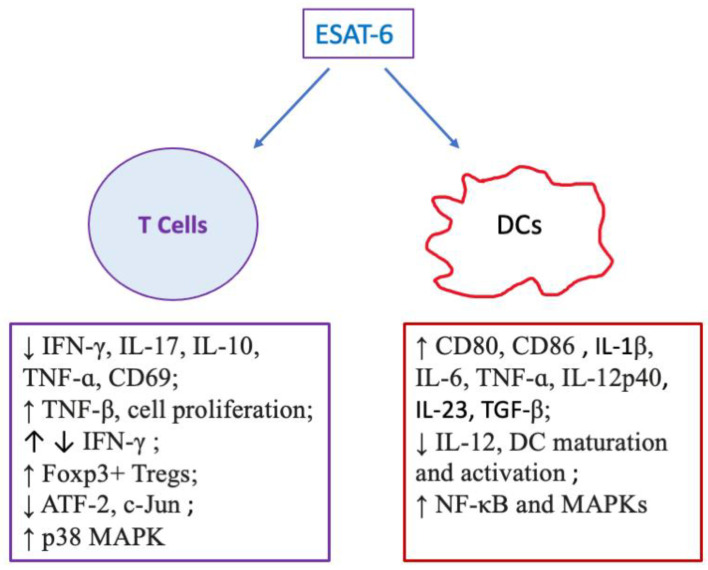
ESAT-6 regulates T cells and DCs via acting on their signal pathways. ESAT-6 largely inhibits T cell activation via suppressing their proliferation and production of critical cytokines IFNγ, IL-17, and TNFα. Moreover, it enhances p38 MAPK while reducing ATF-2 and c-JUN pathways in T cells. Further, ESAT-6 promotes FoxP3^+^ Treg generation. On the other hand, ESAT-6 plays dual roles in regulating DCs. It increases expression of CD80/CD86 in DCs and their production of IL-1, IL-6, IL-12, IL-23, and TNFα through NFκB and MAPK pathways while reducing IL-12 expression and DC maturation. (“↑” denotes increasing, while “↓” indicates decreasing).

Taken together, ESAT-6 either inhibits or promotes T cell activation/function, possibly depending on the experimental contexts, T cell subpopulation used, and the conformational state of this protein (Figure 2). Although the observed suppression of αβ T cell activation and IFN-γ expression in several models indicates that ESAT-6 could be exploited to suppress T-cell-mediated autoimmunity or alloimmunity, it is primarily or traditionally considered as a virulence factor participating in TB immunopathology.

## 5. Anti-Inflammatory Effects of ESAT-6 and Its Potential to Suppress Allograft Rejection

Although ESAT-6 plays dual roles in modulating various immune cells, previous studies have demonstrated its inhibitory effects on some immune cells as well as anti-inflammatory properties under certain circumstances. ESAT-6 has been reported to inhibit inflammatory MyD88/NFkB signaling pathways in macrophages by binding to their TLR2 [46]. It also suppressed the enzyme activity of the matrix metalloproteinase-9 (MMP-9) and reduced the expression of COX-2 and iNOS in RAW 264.7 macrophages activated by LPS [44]. Moreover, it downregulated the expression of some proinflammatory cytokines in macrophages via regulating miR-222-3p [48]. More importantly, it can inhibit the activation of T cells and their production of IFNγ [59]. Therefore, this evidence suggests that ESAT-6 could potentially serve as an anti-inflammatory or immunosuppressive drug in some settings of diseases, at least as a complementary measure, warranting further investigation into its therapeutic applications.

On the other hand, we have recently demonstrated that ESAT-6 can moderately suppress allograft rejection in a murine model and is even more effective when combined with rapamycin, but not cyclosporine [18]. We revealed that ESAT-6 suppressed murine skin and heart allograft rejection, attenuated CD3^+^ T cell infiltration, and increased the percentage of Foxp3^+^ Tregs in vivo. ESAT-6 elevated the frequency of CD4^+^Foxp3^+^ Tregs, while it reduced the frequency of Th1 and effector T cells in peripheral lymphoid organs after transplantation. Interestingly, ESAT-6 induced CD4^+^Foxp3^+^ Tregs from naive CD4^+^CD25^−^ T cells via IĸBα/c-Rel signaling, whereas suppression of c-Rel signaling abolished the induction of Tregs [18]. Furthermore, it inhibited CD4^+^CD25^−^ T cell proliferation. However, ESAT-6 did not interfere with Dendritic cell maturation. Taken together, ESAT-6 inhibited allograft rejection by inducing CD4^+^Foxp3^+^ Tregs via acting on IĸBα/c-Rel signaling. Therefore, it is necessary to determine if ESAT-6 also suppresses human graft rejection in the future.

It is necessary to emphasize that these potential anti-inflammatory and immunosuppressive effects of ESAT-6, especially in context of transplantation, are only based on preliminary and preclinical evidence. The transformation of ESAT-6 from a virulence factor in TB infections to a therapeutic medication would require more extensive investigation, including extensive toxicological assessments, pharmacokinetic analysis, and rigorous efficacy trials in advanced animal models prior to any clinical development. Therefore, it is essential to primarily demonstrate the efficacy and safety of ESAT-6 in regulating human immune responses in vitro and in humanized mouse models before any clinical trial can be considered.

## 6. Conclusions and Future Directions Beyond Tuberculosis

As an immunodominant antigen and main virulence protein of Mtb, ESAT-6 modifies innate and adaptive immunity. Special attention has been paid to the research and development of diagnostic methods and vaccines targeting ESAT-6 in the last 20 years [82,83]. A deeper understanding of the interaction between ESAT-6 and host immune system will help control and cure tuberculosis. Nevertheless, important controversial questions about the exact effects of ESAT-6 on the immune system remain to be answered. Its immunomodulatory effects are much more complex than originally thought in TB study. Although significant progress has been made, the mechanisms underlying the effects of ESAT-6 on various immune cells are still not fully understood. ESAT-6 was reported to not only augment but also inhibit the activation and function of macrophages, DCs and T cells. The discordant results and contradictory influences of ESAT-6 on different immune cells may be attributed to several factors: (1) the conformational states of this protein, which can alter binding affinity of its receptor and functional results [84]; (2) the experimental context or condition, including the cell types or subpopulations used (e.g., naive vs. memory T cells, M1 vs. M2 macrophages), the source of ESAT-6 (e.g., secreted from bacteria vs. recombinant protein), and its protein concentration; (3) the diverse functions of receptors like TLR2 and TLR4 on different immune cells, which can transduce contrary signaling (e.g., NF-κB inhibition in macrophages vs. co-stimulation in T cells); (4) the complex immunological environment, such as the existence of different cytokines or pathogens, which may change the overall response. Thus, more attention is needed to uncover the novel mechanisms underlying the action of ESAT-6 and other virulence factors.

In addition to its role in TB infections, the immunosuppressive properties of ESAT-6 indicate a potential capacity to regulate autoimmunity/alloimmunity or suppress transplant rejection. We have recently shown that ESAT-6 itself can moderately inhibit allograft rejection by promoting Treg generation. However, this potential application is still in its early stage and only represents a forward-looking perspective, although the promising preclinical data deserves further investigation into the therapeutic effects of ESAT-6 on autoinflammatory diseases and allograft survival. Future research should give priority to its safety profile and efficacy in more complicated biological networks and animal models before its therapeutic potential can be practically assessed in humans. Studying the structure–function relationship of ESAT-6 could also contribute to the successful engineering of new synthetic peptides or analogs that preserve its immunosuppressive functions without virulence properties. In summary, although ESAT-6 provides a novel approach to immune regulation, there is a long way to go from a bacterial virulence factor to a potential therapeutic drug.

## Figures and Tables

**Table 1:** Immunoregulatory effects of ESAT-6 on macrophages via different signal pathways.

Cell Type	Origin	Effects	Refs.
Macrophages	Human blood	↑ Fusion of lysosomes and phagosomes, ↑ phagosome rupture	[19]
	Human blood	↓ IL-12, CD80, CD86, IRF5, c-MAF, IL-10, IL-6, CXCL10, CXCL1; ↑ M1 phenotype at the primo-infection, ↑ M2 phenotype at a later stage of the infection	[27]
	Murine bone marrow	↑ TNF-ɑ, MCP-1, IL-1β, IL-6, STAT3 activation	[28,29,30]
	Murine bone marrow	↑ iNOS/NO, E-cadherin, junction plakoglobin, ZO1, desmoplakin, desmoglein3 and catenin proteins; ↓ H3K27 trimethylation; ↑ epithelioid macrophages	[31]
	Murine bone marrow	↑ Cell apoptosis, cleaved caspase-9 and -3, Bim activation, ROS generation, MAPKs phosphorylation	[32]
	THP-1 cells	↑ IL-1β, glucose uptake, DHAP, AcCoA, lipid bodies, foamy macrophage, the activation of NLRP3/ASC inflammasome	[26,33]
	THP-1 cells	↑ ROS, HIF1a, NLRP3 activation, phagocytosis activity, glucose metabolism, cell necrosis, lysosomal permeabilization	[34,35]
	THP-1 cells	↓ NO, ROS, NO synthase activity, MHC-I-β2M complexes on cell surface, class I Ag presentation	[36,37]
	THP-1 cells	↑ M1 polarization, activation of TLR4/MyD88/NF-κB pathway, cell apoptosis within 24h; ↑ M2 phenotype at 36h post-treatment, ↓ TLR4/MyD88/NF-κB pathway	[38]
	THP-1 cells	↑ miR-30a-3p; ↓ miR-30a-5p, autophagy	[39]
	RAW264.7 cells	↑ BAT3, TNF-ɑ, MCP-1, caspase-1, -3, -5, -7 and -8, miR-155, cell apoptosis, NAPDH-ROS-JNK/p38-NF-kB pathway	[40,41,42,43]
	RAW264.7 cells	↓ COX-2, iNOS, NO, ROS, p65 transactivation, expression and enzyme activity of MMP-9;	[44,45]
	RAW264.7 cells	↑ Activation of Akt; ↓ interaction between MyD88 and IRAK4, NF-ĸB activation	[46]
	RAW264.7 cells	↑ ERK1/2 phosphorylation in the cytoplasm; ↓ ERK1/2 phosphorylation in the nucleus, C-myc	[47]
	RAW264.7 cells	↓ IL-1β, IL6, TNFa, p-p65, miR-222-3p; ↑ PTEN	[48]
	Murine peritoneal cavity	↓ NO and ROS; ↑ apoptosis and necrosis	[49]
	Murine peritoneal cavity	↑ HFE in endoplasmic reticulum; ↓ HFE-TFR1 complex on cellular surface; ↑ holotransferrin-regulated iron uptake	[50]
	Murine bone marrow, peritoneal cavity, MH-S cells	↑ IFN-β; ↑ activation of TBK1 and IRF3	[51]
	Murine abdominal cavity, RAW264.7 cells	↓ LC3II and SQSTM1 degradation; ↓ autophagy flux; ↑ mTOR activity	[52]
	J774 A.1 cell	↑ Expression and activity of SOD-2	[53]
	Murine peritoneal cavity	↑ COX-2, PI3K and MAPK pathway	[54]

(“↑” denotes increasing, while “↓” indicates decreasing).

**Table 2:** Immunoregulatory effects of ESAT-6 on other immune cells.

Cell Type	Origin	Effects	Ref.
DCs	Murine bone marrow	↑ IL-6, TGF-β	[55]
	Murine bone marrow	↑ CD80, CD86 and MHC-II, IL-6, TNF-ɑ and IL-12p40, activation of NF-κB and MAPKs	[56]
	Human blood	↓ IL-12, DC maturation and activation; ↑ IL-1β and IL-23	[57]
Neutrophils	Human blood	↑ Intracellular Ca_2_+ overload, neutrophil extracellular traps, necrosis	[58]
T cells	Human blood	↓ IFN-γ, IL-17, TNF-ɑ, CD69, ATF-2, c-Jun	[57]
	Human blood	↓ IFN-γ, IL-10, IL-17; ↑ activation of p38 MAPK	[59]
	Human blood	↑ TNFβ in CD4+ T cells and IFN-γ in CD8+ T cells	[60]
	Human blood	↑ Cell activation and proliferation	[61]
	Murine skin/spleen, and lymph nodes	↓ CD3+ T cells in allografts, Th1, CD4+/CD8+ effector T cells in spleen and lymph nodes; ↑ Foxp3+ Treg in an allograft, spleen and lymph nodes; ↑ CD4+Foxp3+ Tregs differentiation, IĸBα/c-Rel signaling pathway in vitro; ↓ CD4+CD25− T cell proliferation in vitro	[18]

(“↑” denotes increasing, while “↓” indicates decreasing).

## Data Availability

Not applicable.

## Abbreviations

The following abbreviations are used in this manuscript:

ESAT-6	early secreted antigenic target of 6 kDa
COX-2	cyclooxygenase-2
DC	dendritic cell
BMDM	bone marrow-derived macrophage
MCP-1	monocyte chemoattractant protein-1
Mtb	mycobacterium tuberculosis
NO	nitric oxide
ROS	reactive oxygen species
